# Body Shape Indices in Adolescents Based on the 2009–2012 Korea National Health and Nutrition Examination Survey

**DOI:** 10.3390/children8100894

**Published:** 2021-10-07

**Authors:** Byung Ok Kwak, Jisun Lim, Sochung Chung

**Affiliations:** 1 Department of Pediatrics, Hallym University Kangnam Sacred Heart Hospital, Seoul 07441, Korea; qquack00@hanmail.net; 2 Research Institute of Basic Science, Seoul National University, Seoul 08826, Korea; swanjslim@gmail.com; 3 Department of Pediatrics, Konkuk University Medical Center, Konkuk University School of Medicine, Seoul 05030, Korea

**Keywords:** body shape index, body mass index, waist circumference, adolescent, obesity

## Abstract

A Body Shape Index (ABSI) is a recently proposed index for standardizing waist circumference (WC) for body mass index (BMI) and height in adults, using 2/3 and 1/2 as scaling exponents, respectively. However, ABSI has limited applicability to children and adolescents, as the relationship between height and weight changes with age and varies according to sex. This study aimed to investigate whether ABSI can be applied to adolescents and to analyze the relationships among BMI, WC, height, weight, and body shape index (BSI) in Korean adolescents. The data of 1023 adolescents aged 10–19 years from the 2009–2012 Korea National Health and Nutrition Examination Survey were collected. Body measurements (height, weight, WC, and BMI) were analyzed to estimate the BSI using log-linear regression. The scaling exponents for standardizing WC for weight and height were estimated according to age (per year) and sex. The scaling exponents for standardizing WC for weight and height were 0.698 and −1.090 for boys and 0.646 and −0.855 for girls, respectively. The exponents also differed according to age. BSI was negatively correlated with height, weight, and BMI in boys and girls, and these correlations differed in direction from those in adults. ABSI cannot be applied to adolescents. In adolescents, the BSI is dependent on age and sex and is associated with growth and puberty. Further studies are required to evaluate the association between BSI and other biomarkers, to improve its applicability as a parameter for predicting the risk of chronic diseases in adolescents.

## 1. Introduction

The prevalence of obesity among children and adolescents has increased worldwide [1]. Pediatric obesity is a public health concern because it is considered to be associated with a wide range of diseases, including type 2 diabetes mellitus, dyslipidemia, hypertension, nonalcoholic fatty liver disease, and obstructive sleep apnea in childhood [2,3,4,5,6]. Moreover, obesity in childhood is also linked to a higher risk of obesity and comorbidities in adulthood [7,8,9].

An increasing prevalence of overweight and obesity in the pediatric population has been reported worldwide [10,11]. The prevalence of obesity among children aged 2–19 years in the United States increased from 10.0% in 1988–1994 to 17.2% in 2013–2014 [10]. In Korea, a recent study showed that the prevalence of obesity increased from 8.7% in 2007 to 15.0% in 2017 in children aged 6–18 years, and from 8.6% in 2001 to 9.8% in 2017 in children aged 2–18 years, based on the National School Health Examination and Korea National Health and Nutrition Examination Survey (KNHANES) data [11].

Body mass index (BMI) has been considered an indicator of body fatness and is widely used as a screening method for obesity [12]. However, BMI cannot differentiate body fat from lean mass and cannot provide information about the distribution of fat in the body. Although waist circumference (WC) is now a widely used measure of abdominal obesity, it has limitations in complementing BMI in predicting mortality and health risks because of its high collinearity with weight and BMI [13,14]. To differentiate between obesity and abdominal obesity, Krakauer and Krakauer developed a new anthropometric measure, called A Body Shape Index (ABSI), which estimates body shape using measurements of height, weight, and WC, using the data of 14,105 adults who participated in the National Health and Nutrition Examination Survey (NHANES) in 1999–2004 [13]. In their study, ABSI did not correlate with BMI or WC but was considered a better predictor of premature mortality risk than BMI and WC.

However, in another study, ABSI unexpectedly showed a negative association with blood pressure in Portuguese adolescents aged 10–17 years [15]. Therefore, it is considered that the body shape of adolescents differs from that of adults and differs according to age. Recently, Xu et al. analyzed 562 adolescents (aged 10–17 years) from the 2009 China Health and Nutrition Survey and proposed the “ABSI-adolescents” formula using new scaling exponents for standardizing WC for BMI and height [16].

We hypothesized that ABSI obtained from adults cannot be applied to adolescents and examined the age- and sex-specific body shape index (BSI) in adolescents. The aim of the present study was to estimate the scaling exponents for standardizing WC for weight and height in Korean adolescents and to analyze the relationships among BMI, WC, height, weight, and BSI.

## 2. Materials and Methods

### 2.1. Study Design and Population

This study was performed using data from the 2009–2012 KNHANES. The KNHANES is a nationwide cross-sectional survey conducted annually by the Korea Centers for Disease Control and Prevention [17]. Its target population comprises nationally representative, noninstitutionalized civilians aged ≥ 1 year in Korea, selected using a stratified, multistage, clustered probability sampling method. The KNHANES is composed of a health interview, health examination survey, and nutrition survey. All surveys were conducted with the participants’ consent, and data from the KNHANES were obtained in a fully anonymized and de-identified manner. Therefore, this study was exempt from the requirement for approval by the Konkuk University Medical Center Institutional Review Board.

### 2.2. Study Variables

Body measurements, including height, weight, and WC, were obtained by trained health technicians following standardized procedures. Height was measured to the nearest 0.1 cm with a wall-mounted stadiometer. Weight was measured, with the participants in light clothing, to the nearest 0.1 kg on a digital scale. WC was measured using a steel tape, at the midpoint between the lower border of the rib cage and the iliac crest, to the nearest 0.1 cm. BMI was defined as weight (kg) divided by height squared (m^2^).

We performed linear least-squares regression on log (WC) as a function of log (weight) and log (height). The coefficients from log-log regression were the scaling ex-ponents for weight and height that were used to calculate the BSI, as follows:

log (WC) = b_0_ + b_1_ log (weight) + b_2_ log (height)

WC ∝ weight (b_1_) height (b_2_)

BSI ≡ WC weight (b_1_) height (b_2_)

While ABSI was developed by Krakauer and Krakauer based on the data of adults from the NHANES, BSI was determined as an adapted formula for adolescents by age based on the data of adolescents to estimate new scaling exponents for standardizing WC for weight and height. To verify our hypothesis, we applied ABSI to our study population, and compared it with BSI.

### 2.3. Statistical Analysis

Descriptive analyses were performed to characterize the samples. The normality of the distribution of each variable was tested using the Shapiro–Wilk test, and logarithmic transformation was used when needed. Mean values were compared using an independent *t*-test. Linear least-squares regression models were constructed with log (WC) as a function of log (height) and log (weight). Pearson’s correlation coefficients were used to estimate the associations among the different variables (height, weight, BMI, WC, and BSI). In correlations analysis, the statistical tests were two-sided, with an alpha level of 0.05. All analyses were conducted using R software (version 3.5.3). Scatter plots for the study variables were created using MATLAB R2020a software.

## 3. Results

A total of 1023 adolescents was included in the analysis. Table 1 shows the anthropometric profiles of the participants. The mean (± standard deviation [SD]) age of the participants was 13.9 ± 2.8 years, and the number of boys and girls was 542 (53.0%) and 481 (47.0%), respectively. The mean (±SD) WC, weight, and height were 70.7 ± 10.5 cm, 55.8 ± 15.5 kg, and 162.4 ± 13.2 cm in boys and 66.8 ± 8.3 cm, 50.0 ± 10.5 kg, and 157.2 ± 8.2 cm in girls, respectively. The mean BSI and ABSI by age groups were also presented.

In the 1023 adolescents, the BSI was calculated using the above equations [13], with 0.678 and −0.985 as the scaling exponents for weight and height, respectively (R^2^ = 0.842). The estimated exponents for weight and height were 0.698 and -1.090 for boys (R^2^ = 0.876) and 0.646 and −0.855 for girls (R^2^ = 0.794), respectively (Table 2). The estimated values of b_1_ and b_2_ for BSI also varied according to age in adolescents (Table 3).

**Figure 1:**
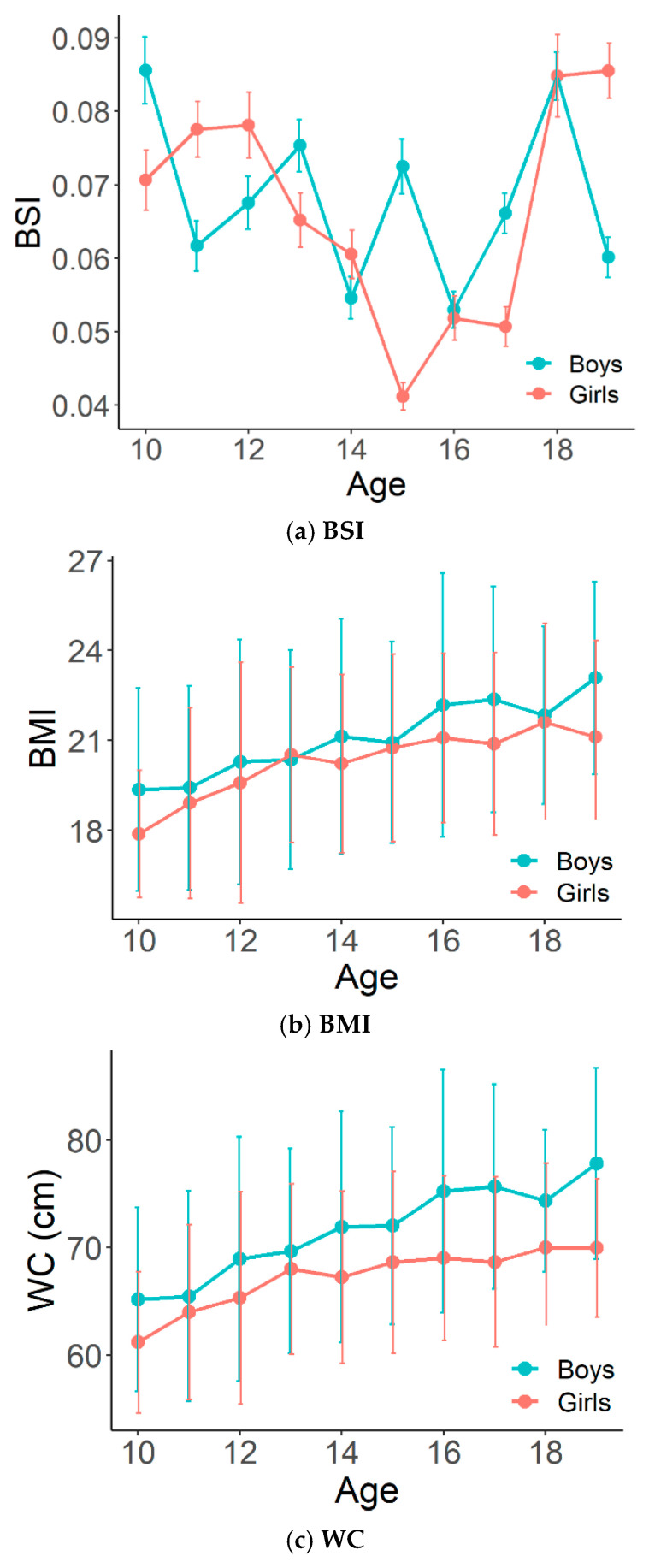
Means and standard deviations of BSI, BMI, and WC among boys and girls according to age. BSI body shape index, BMI body mass index, WC waist circumference.

**Figure 2:**
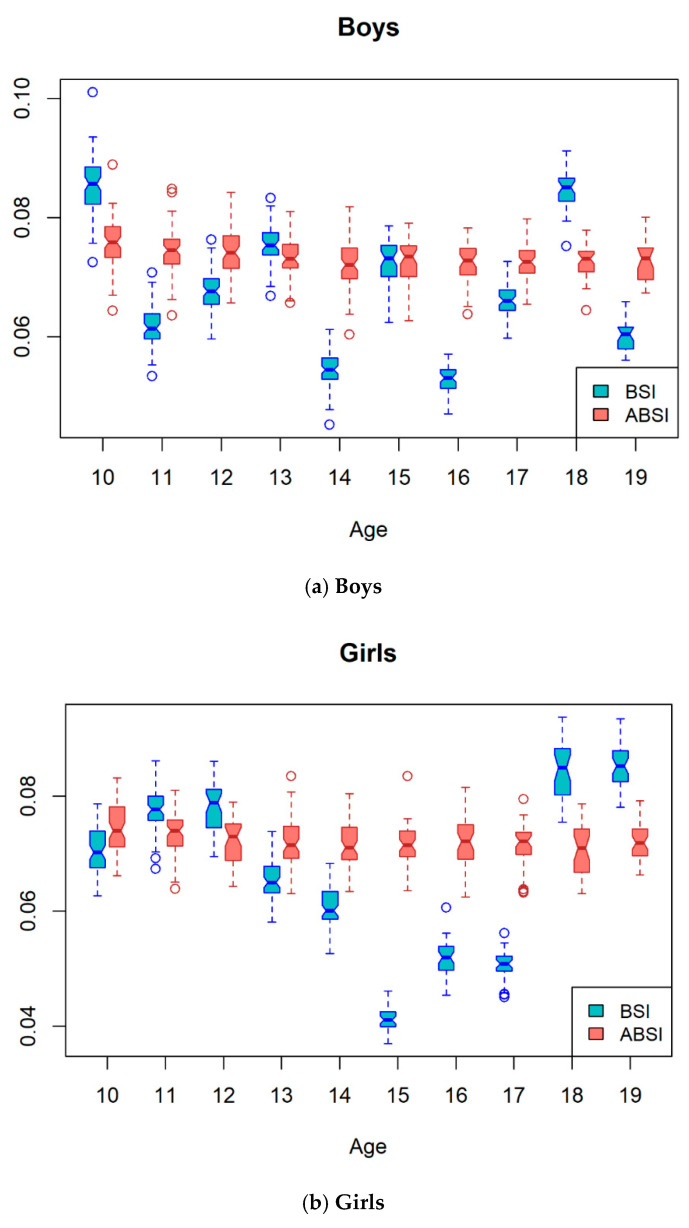
Comparison between mean BSI and ABSI among boys and girls according to age. BSI body shape index, ABSI a body shape index.

The sex-specific mean BSI showed a different pattern (Figure 1). The mean BSI was significantly different between boys and girls, except for the 16 and 18 years of age groups. The mean BSI in boys remained relatively constant with a slight increase and decrease, whereas the mean BSI in girls increased up to age 12 years, then decreased, and increased again after age 15 years. The mean peak BSI was observed at age 10 and 19 years in boys and girls, respectively. The lowest BSI was observed in girls aged 15 years. In contrast to the BSI, the mean BMI and WC showed increasing patterns according to age in both boys and girls, and were higher in boys than in girls. Comparison between the mean BSI and ABSI among boys and girls according to age groups is presented in Figure 2. While the mean ABSI remained relatively constant in boys and girls, the mean BSI varied by age and gender.

**Figure 3:**
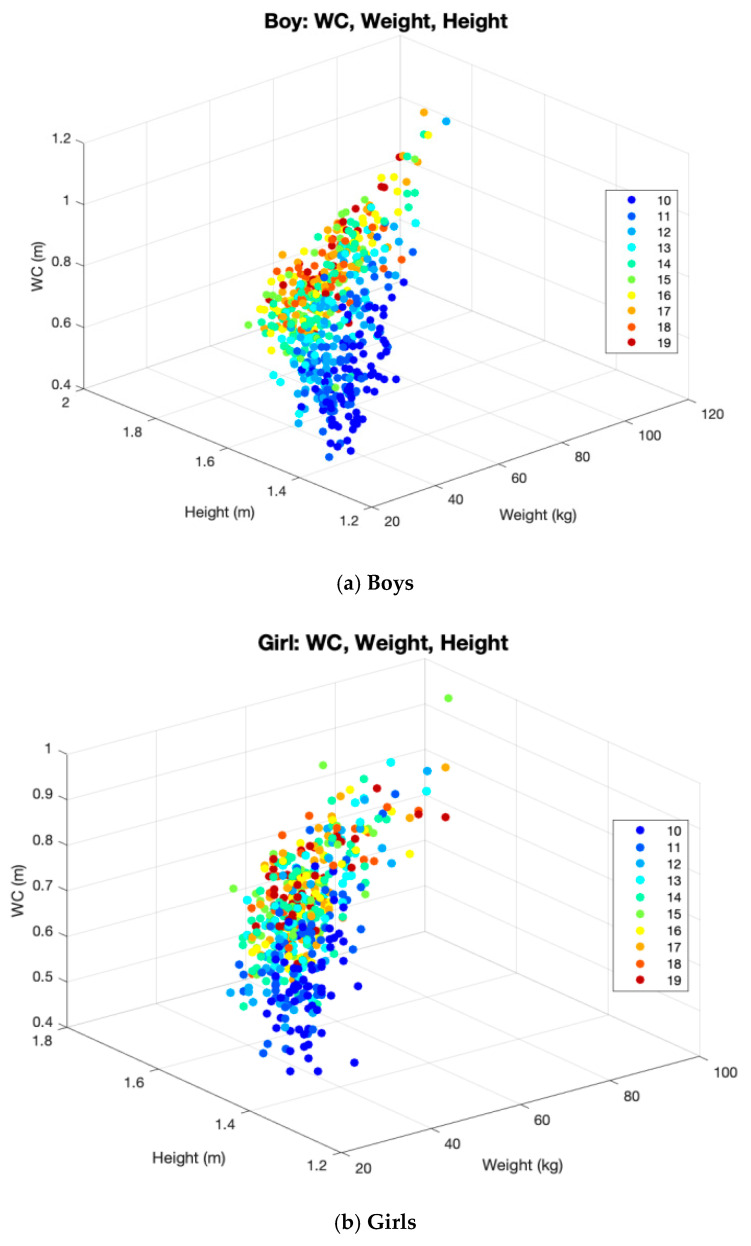
Scatter plot of the relationships among height, weight, and WC according to age in boys and girls. WC waist circumference.

The relationships among height, weight, and WC according to age in boys and girls are presented in Figure 3.

Table 4 shows the correlation coefficients between BSI and other anthropometric variables and biomarkers alongside with ABSI in boys and girls. BMI and WC were positively correlated with height and weight. BSI was negatively associated with height and weight in both boys and girls, and negatively associated with BMI in boys. No correlation was found between BSI and WC. ABSI had a negative correlation with height and weight, and a positive correlation with WC in both boys and girls.

However, in a subgroup analysis according to age and sex, BSI had a positive correlation with WC in all ages but showed no correlation with height, weight, and BMI (Table 5). On the other hand, ABSI did not correlate with WC in the 12 and 19 years of age groups of girls.

## 4. Discussion

We estimated scaling exponents for weight and height to calculate the BSI in adolescents using data from the KNHANES. The exponents differed according to age and sex. The BSI in adolescents is age and sex dependent and is associated with growth and puberty.

An increase in pediatric obesity is a global health issue [1,10,11,18]. Behavioral factors, such as diet, physical activity, and socioeconomic status can affect the obesity epidemic [11,18]. Furthermore, pediatric obesity becomes an even bigger health concern from climate change because of a strong relationship between temperature and BMI [18].

BMI is a widely accepted measure of general obesity; however, it does not distinguish between muscle and fat accumulation and does not provide information on fat distribution [12,16]. Therefore, alternative indices that differentiate different types of fat distribution have been proposed, including WC, waist-to-hip ratio, and waist-to-height ratio. Recently, ABSI was introduced by Krauer and Krauer using data from >14,000 adults from the NHANES [13]. However, whether the same scaling exponents can be used to properly standardize WC for BMI and height in adolescents is unclear [15,16,19]. Different scaling exponents can be applied across different age ranges.

A Portuguese study showed a negative correlation between ABSI and systolic and diastolic blood pressure, and this negative correlation may be due to the application of inappropriate scaling exponents in adolescents [15]. Therefore, Xu et al. proposed the ABSI-adolescents formula using different scaling exponents for standardizing WC for BMI and height among Chinese adolescents, and this formula was more strongly associated with glycated hemoglobin and pre-diabetes, but not with blood pressure, than BMI [16]. Moreover, ABSI-adolescents, but not ABSI, were found to be related to the adiponectin-to-leptin ratio and to markers of glucose metabolism among 197 Brazilian adolescents aged 14–18 years [19]; however, even ABSI-adolescents did not show a stronger association than BMI and WC. Therefore, we examined the age- and sex-specific BSI in adolescents. The inversely negative correlation between ABSI and blood pressure, or a weaker correlation to markers of glucose metabolism compared to BMI or WC, was created by an application of scaling exponents derived from an adult population or the same exponents, which were not age- and sex-specific, to adolescents. Although the association between BSI and cardiometabolic risks was not investigated, the correlation between BSI and other anthropometrics, predictors of metabolic health outcomes was analyzed in this study. More intensive research comparing BSI and other metabolic biomarkers will be conducted to confirm its clinical significance and applicability in adolescents.

In previous studies, the mean BSI steadily increased from midlife to old age in adults [13,14]. Conversely, in our study, the mean BSI in adolescents showed different change patterns, with significant variability according to growth and pubertal development. The variability in BSI was higher in girls than in boys across all ages. In boys, the BSI relatively slightly increased and decreased. In girls, BSI significantly increased, then decreased after age 12 years, and increased again thereafter. Meanwhile, the mean BMI and WC showed increasing patterns with age in both boys and girls, and were higher in boys than in girls. This means that BMI and WC seem to increase with age, whereas BSI based on WC adjusted for weight and height does not, as fat distribution changes after the peak height velocity in adolescents. Moreover, BSI is derived from its dependence on height according to age and sex.

In our present study, the scaling exponent for height in Korean adolescents was higher than that in American and Korean adults [13,14]. This means that as height increases, WC increases more in adolescents than in adults. The ABSI obtained from the adult population is difficult to apply to the pediatric population because the annual height, weight, and BMI velocity change with age, and fat distribution in adolescents varies according to several factors, including age, sex, pubertal development, and nutritional status [12]. Body composition changes do not show similar patterns between adolescents and adults [19]. Unlike in adulthood, weight and height during adolescence increase with age in different patterns according to sex, providing specific weight-height relationships that change over time. As shown in our results comparing ABSI and BSI, the mean ABSI remained relatively constant, whereas the mean BSI changed by age and gender. The variability according to age and different change patterns between boys and girls of the mean BSI is associated with growth and pubertal development. This means that BSI based on WC adjusted for weight and height is dependent on growth and fat distribution changes after the peak height velocity in adolescents. Moreover, BSI had a positive correlation with WC in all age groups, but ABSI had no correlation with WC in some age groups. Therefore, it is not appropriate to apply ABSI to adolescents, because ABSI does not reflect the physiologic changes of adolescence by age and gender. In adolescents, BSI obtained using different scaling exponents depending on age and sex is needed. The strength of our study is that we estimated BSI according to age (per year) and sex in adolescents considering growth and pubertal development.

This study had some limitations. First, we estimated the scaling exponents for BSI only in the Korean adolescent population. Thus, the results should be confirmed in pediatric groups of other ethnicities. Second, we could not investigate the relationship between BSI and obesity-related mortality or morbidity in this study. In addition, we could not differentiate between fat mass and fat-free mass and could not evaluate the correlation between BSI and muscle mass.

## 5. Conclusions

The ABSI used in the adult population cannot be applied to adolescents. BSI in adolescents is age and sex dependent and is associated with growth and puberty. Further studies are required to confirm our results in other ethnic groups of different ages, and to evaluate the association between BSI and other biomarkers to improve its applicability as a parameter for predicting the risk of chronic diseases in adolescents.

## Figures and Tables

**Table 1:** Characteristics of the participants according to age.

Age (Years)	*n*	Height (cm)	Weight (kg)	BMI (kg/m^2^)	WC (cm)	BSI	ABSI
**Boys**							
10	74	142.4 ± 6.6 *	39.5 ± 8.6	19.4 ± 3.4 *	65.1 ± 8.6 *	0.0856 ± 0.0045 *	0.0760 ± 0.0041
11	68	148.9 ± 7.2 *	43.4 ± 10.1	19.4 ± 3.4	65.5 ± 9.8	0.0617 ± 0.0034 *	0.0743 ± 0.0042
12	70	156.7 ± 7.4	50.3 ± 13.2	20.3 ± 4.1	68.9 ± 11.4	0.0676 ± 0.0036 *	0.0742 ± 0.0042
13	53	163.3 ± 7.5 *	54.6 ± 11.6	20.4 ± 3.7	69.6 ± 9.5	0.0754 ± 0.0035 *	0.0733 ± 0.0035
14	71	169.9 ± 5.9 *	61.1 ± 12.5 *	21.1 ± 3.9	71.9 ± 10.8 *	0.0546 ± 0.0028 *	0.0723 ± 0.0039
15	45	171.4 ± 6.8 *	61.8 ± 12.0 *	20.9 ± 3.4	72.0 ± 9.2	0.0546 ± 0.0028 *	0.0726 ± 0.0037
16	44	173.7 ± 4.4 *	66.9 ± 13.5 *	22.2 ± 4.4	75.2 ± 11.3 *	0.0530 ± 0.0025	0.0725 ± 0.0035
17	53	173.9 ± 5.4 *	67.8 ± 13.3 *	22.4 ± 3.8 *	75.6 ± 9.5 *	0.0661 ± 0.0027 *	0.0724 ± 0.0030
18	39	172.6 ± 6.1 *	64.9 ± 8.3 *	21.8 ± 3.0	74.3 ± 6.6 *	0.0848 ± 0.0033	0.0727 ± 0.0028
19	25	174.4 ± 6.1 *	70.4 ± 12.1 *	23.1 ± 3.2 *	77.8 ± 8.9 *	0.0601 ± 0.0027 *	0.0728 ± 0.0033
Total	542	162.4 ± 13.2 *	55.8 ± 15.5 *	20.8 ± 3.8 *	70.7 ± 10.5 *	0.0683 ± 0.0115 *	0.0735 ± 0.0039
**Girls**							
10	64	144.9 ± 6.7 *	37.8 ± 6.5	17.8 ± 2.1 *	61.2 ± 6.6	0.0707 ± 0.0041 *	0.0744 ± 0.0043
11	61	151.9 ± 7.0 *	43.9 ± 9.6	18.9 ± 3.2	64.0 ± 8.1	0.0776 ± 0.0151 *	0.0734 ± 0.0042
12	39	156.5 ± 6.0	48.3 ± 11.7	19.6 ± 4.0	65.3 ± 9.9	0.0781 ± 0.0045 *	0.0721 ± 0.0042
13	61	158.5 ± 5.3 *	51.7 ± 9.1	20.5 ± 2.9	68.0 ± 7.9	0.0652 ± 0.0037 *	0.0722 ± 0.0041
14	59	160.4 ± 6.3 *	52.2 ± 8.8 *	20.2 ± 3.0	67.2 ± 8.0 *	0.0605 ± 0.0033 *	0.0716 ± 0.0040
15	44	162.3 ± 4.9 *	54.6 ± 8.7 *	20.7 ± 3.1	68.6 ± 8.5	0.0412 ± 0.0019 *	0.0714 ± 0.0036
16	42	159.6 ± 4.6 *	53.7 ± 7.6 *	21.1 ± 2.8	69.0 ± 7.7 *	0.0519 ± 0.0030	0.0717 ± 0.0043
17	40	161.7 ± 6.0 *	54.7 ± 8.5 *	20.9 ± 3.0 *	68.7 ± 7.9 *	0.0507 ± 0.0027 *	0.0713 ± 0.0039
18	31	162.5 ± 5.2 *	56.8 ± 8.1 *	21.6 ± 3.3	70.0 ± 7.8 *	0.0849 ± 0.0056	0.0710 ± 0.0047
19	40	161.8 ± 5.0 *	55.2 ± 7.8 *	21.1 ± 3.2 *	70.0 ± 8.3 *	0.0855 ± 0.0037 *	0.0722 ± 0.0033
Total	481	157.2 ± 8.2 *	50.0 ± 10.5 *	20.1 ± 3.2 *	66.8 ± 8.3 *	0.0664 ± 0.0140 *	0.0723 ± 0.0041

Data are presented as mean ± standard deviation. BMI body mass index, WC waist circumference, BSI body shape index, ABSI a body shape index. * Statistically significantly different between boys and girls (*p* < 0.05).

**Table 2:** Regression coefficients of the relationships among log of waist circumference (m), log of weight (kg), and log of height (m).

	Coefficients			R^2^
	Intercept (b_0_)	Log (Weight) (b_1_)	Log (Height) (b_2_)	
**All** (*N* = 1023)	6.551 *	0.678 *	−0.985 *	0.842
**Boys** (*n* = 542)	7.014 *	0.698 *	−1.090 *	0.876
**Girls** (*n* = 481)	6.009 *	0.646 *	−0.855 *	0.794

* *p* < 0.001.

**Table 3:** Regression coefficients of the relationships among log of waist circumference (m), log of weight (kg), and log of height (m) according to age.

Sex	Age (Years)	Coefficients			R^2^
		Intercept (b_0_)	Log (Weight) (b_1_)	Log (Height) (b_2_)	
**Boys**	10	−2.4594 *	0.6393 *	−0.8873 *	0.833
	11	−2.7872 *	0.7239 *	−0.9037 *	0.852
	12	−2.6958 *	0.7510 *	−1.3563 *	0.882
	13	−2.5866 *	0.6986 *	−1.1493 *	0.878
	14	−2.9089 *	0.7374 *	−0.8494 *	0.861
	15	−2.6254 *	0.6494 *	−0.6994	0.820
	16	−2.9387 *	0.7075 *	−0.5741	0.896
	17	−2.7174 *	0.6883 *	−0.8325 *	0.884
	18	−2.4683 *	0.6271 *	−0.8147 *	0.807
	19	−2.8122 *	0.6978 *	−0.7272	0.840
**Girls**	10	−2.6518 *	0.6893 *	−0.9149 *	0.704
	11	−2.5578 *	0.6296 *	−0.6316	0.846
	12	−2.5512 *	0.6316 *	−0.7089	0.841
	13	−2.7321 *	0.6556 *	−0.5172	0.747
	14	−2.8048 *	0.6781 *	−0.5720	0.785
	15	−3.1910 *	0.7498 *	−0.3796	0.847
	16	−2.9609 *	0.6909 *	−0.3457	0.717
	17	−2.9835 *	0.7334 *	−0.6779	0.770
	18	−2.4692 *	0.6221 *	−0.8295	0.639
	19	−2.4595 *	0.5955 *	−0.5926	0.765

* *p* < 0.001.

**Table 4:** Correlations among height, weight, BMI, WC, BSI, and ABSI in adolescents.

		Height	Weight	BMI	WC	BSI	ABSI
**All**	Height	1	0.7565 *	0.3329 *	0.4702 *	−0.48 *	−0.1609 *
	Weight		1	0.8596 *	0.8716 *	−0.3455 *	−0.0805 *
	BMI			1	0.9036 *	−0.1471 *	−0.0095
	WC				1	−0.0103	0.3402 *
	BSI					1	0.6084 *
	ABSI						1
**Boys**	Height	1	0.7658 *	0.331 *	0.4531 *	−0.3576 *	−0.2761 *
	Weight			0.8508 *	0.8705 *	−0.2628 *	−0.1313 *
	BMI			1	0.9274 *	−0.104 *	0.0213
	WC					−0.0346	0.2943 *
	BSI					1	0.4389 *
	ABSI						1
**Girls**	Height	1	0.6888 *	0.3048 *	0.4361 *	−0.2142 *	−0.0926 *
	Weight		1	0.8965 *	0.8633 *	−0.1614 *	−0.0989 *
	BMI			1	0.871 *	−0.087	−0.0852
	WC				1	0.0275	0.3631 *
	BSI						0.3386 *
	ABSI						1

BMI body mass index, WC waist circumference, BSI body shape index, ABSI a body shape index. * *p* < 0.05.

**Table 5:** Correlations among height, weight, BMI, WC, and BSI in boys and girls according to age.

Age (Years)		Height	Weight	BMI	WC	BSI	ABSI
10	Height	1	0.634 *	0.271 *	0.368 *	0.000	−0.118
	Weight	0.743 *	1	0.913 *	0.873 *	0.010	−0.132
	BMI	0.294 *	0.855 *	1	0.900 *	0.015	−0.101
	WC	0.414 *	0.791 *	0.820 *	1	0.427 *	0.307 *
	BSI	−0.005	−0.009	0.004	0.542 *	1	0.989 *
	ABSI	−0.021	0.008	0.041	0.566 *	0.999 *	1
11	Height	1	0.697 *	0.356 *	0.472 *	−0.010	0.090
	Weight	0.665 *	1	0.913 *	0.901 *	0.008	0.192
	BMI	0.315 *	0.915 *	1	0.919 *	0.018	0.206
	WC	0.487 *	0.908 *	0.900 *	1	0.373 *	0.543 *
	BSI	-0.002	−0.003	−0.001	0.376 *	1	0.982 *
	ABSI	0.078	−0.038	−0.088	0.317 *	0.990 *	1
12	Height	1	0.701 *	0.443 *	0.448 *	0.006	−0.164
	Weight	0.624 *	1	0.946 *	0.902 *	0.017	0.079
	BMI	0.395 *	0.963 *	1	0.943 *	0.019	0.175
	WC	0.492 *	0.922 *	0.911 *	1	0.344 *	0.466 *
	BSI	0.001	0.035	0.021	0.398 *	1	0.950 *
	ABSI	−0.014	−0.057	−0.082	0.306	0.994 *	1
13	Height	1	0.574 *	0.181	0.266	0.002	−0.218
	Weight	0.576 *	1	0.906 *	0.892 *	0.028	−0.010
	BMI	0.237	0.930 *	1	0.933 *	0.010	0..079
	WC	0.413 *	0.857 *	0.837 *	1	0.354 *	0.384 *
	BSI	0.004	−0.005	−0.002	0.495 *	1	0.970 *
	ABSI	0.176	0.068	0.008	0.534 *	0.985 *	1
14	Height	1	0.371 *	0.040	−0.172	−0.004	0.082
	Weight	0.500 *	1	0.940 *	0.915 *	0.014	0.261 *
	BMI	0.048	0.887 *	1	0.927 *	0.021	0.257 *
	WC	0.297 *	0.868 *	0.848 *	1	0.366 *	0.583 *
	BSI	0.000	−0.004	0.001	0.462 *	1	0.968 *
	ABSI	0.201	0.123	0.041	0.538 *	0.979 *	1
15	Height	1	0.574 *	0.247	0.395 *	0.017	0.085
	Weight	0.272	1	0.933 *	0.906 *	0.047	0.045
	BMI	-0.106	0.926 *	1	0.899 *	0.038	0.006
	WC	0.181	0.928 *	0.885 *	1	0.432 *	0.416 *
	BSI	0.007	0.017	0.006	0.378 *	1	0.996 *
	ABSI	0.356	0.338	0.203	0.620 *	0.906 *	1
16	Height	1	0.162	−0.085	0.051	−0.007	0.160
	Weight	0.355 *	1	0.968 *	0.946 *	0.018	0.218
	BMI	−0.052	0.914 *	1	0.945 *	0.034	0.184
	WC	0.231	0.846 *	0.800 *	1	0.328 *	0.485 *
	BSI	−0.007	0.008	0.001	0.528 *	1	0.972 *
	ABSI	0.251	0.145	0.041	0.614 *	0.965 *	1
17	Height	1	0.496 *	0.182	0.298 *	0.000	0.046
	Weight	0.422 *	1	0.942 *	0.924 *	−0.006	0.090
	BMI	−0.076	0.869 *	1	0.936 *	−0.006	0.085
	WC	0.191	0.870 *	0.858 *	1	0.320 *	0.408 *
	BSI	0.001	−0.015	−0.009	0.434 *	1	0.995 *
	ABSI	0.187	0.207	0.135	0.596 *	0.970 *	1
18	Height	1	0.207	−0.339 *	−0.133	−0.006	−0.017
	Weight	0.133	1	0.848 *	0.844 *	0.008	−0.123
	BMI	−0.306	0.901 *	1	0.875 *	−0.007	−0.127
	WC	−0.131	0.763 *	0.794 *	1	0.440 *	0.320 *
	BSI	0.003	−0.006	−0.001	0.593 *	1	0.991 *
	ABSI	−0.012	−0.010	−0.085	0.518 *	0.995 *	1
19	Height	1	0.560 *	0.184	0.333	−0.009	0.127
	Weight	0.175	1	0.915 *	0.884 *	−0.015	0.135
	BMI	−0.271	0.897 *	1	0.904 *	0.016	0.128
	WC	−0.037	0.863 *	0.856 *	1	0.402*	0.518 *
	BSI	−0.008	−0.015	−0.016	0.454 *	1	0.987 *
	ABSI	0.109	−0.197	−0.245	0.253	0.971 *	1

The right side (above the diagonal) and left side (below the diagonal) for each age represent correlations in boys and girls, respectively. BMI body mass index, WC waist circumference, BSI body shape index, ABSI a body shape index. * *p* < 0.05.

## Data Availability

The data presented in this study are available on request from the corresponding author.
